# Genomic Characterization of Peruvian Creole Goats: Insights into Population Structure and Runs of Homozygosity

**DOI:** 10.3390/ani15172577

**Published:** 2025-09-02

**Authors:** Flor-Anita Corredor, David Godoy-Padilla, Emmanuel Alexander Sessarego, Víctor Temoche-Socola, Miguel Enrique Paredes Chocce, Héctor Escobar Robledo, Máximo Fabricio Ramírez Antaurco, William Burgos-Paz, José Ruiz, Juancarlos Cruz, Henrique A. Mulim, Hinayah Rojas de Oliveira

**Affiliations:** 1 Dirección de Servicios Estratégicos Agrarios, Instituto Nacional de Innovación Agraria (INIA), Lima 15024, Peruinvestigacion_procap@inia.gob.pe (E.A.S.); temochsoc@gmail.com (V.T.-S.); direccion_procap@inia.gob.pe (J.R.); jcruz@inia.gob.pe (J.C.); 2 Estación Experimental Agraria Vista Florida, Instituto Nacional de Innovación Agraria (INIA), Lambayeque 14300, Peru; antaurcomax@gmail.com; 3 Corporación Colombiana de Investigación Agropecuaria-AGROSAVIA, Centro de Investigación Turipaná, Km 13 Vía Montería-Cereté, Montería 230550, Colombia; 4 Department of Animal Sciences, Purdue University, West Lafayette, IN 47907, USAhinayah@purdue.edu (H.R.d.O.)

**Keywords:** Peruvian Creole goats, runs of homozygosity, effective population size, genomic inbreeding, conservation genetics

## Abstract

Peruvian Creole goats (PCGs) represent a unique genetic resource shaped by adaptation to diverse environments and traditional breeding practices. In this study, we performed a genomic analysis of six regional populations (Ancash, Ica, Lambayeque, Lima, Piura, and Tumbes) using high-density SNP genotype data. Principal component analysis revealed a moderate genetic structure, with the Ica population showing clear separation and northern populations exhibiting overlap. Runs of homozygosity were predominantly short, and specific regions on chromosome 6 were shared across populations. Inbreeding coefficients were generally low, with Ancash showing the highest values. Linkage disequilibrium decayed rapidly over genetic distance, especially in Piura, indicating higher genetic diversity. Estimates of effective population size revealed decreasing trends across populations, with Piura maintaining the largest recent population size. These findings offer valuable insights into the population structure of Peruvian Creole goats, providing guidance for conservation and sustainable breeding efforts.

## Simple Summary

Peruvian Creole goats are crucial for rural families in Peru, as they provide milk and meat, in various environments across the country. However, little is known about their genetic characteristics. This study analyzed goats from six regions of Peru using modern genetic tools to understand their relationships. We found that goats from some areas are more genetically distinct, such as those from Ica. We also identified regions in their DNA that may be linked to adaptation to local conditions. These findings are crucial for the protection of this locally adapted animal, as they contribute support to long-term conservation efforts.

## 1. Introduction

Peruvian Creole goats (PCGs) are a genetically and culturally important livestock resource, shaped by centuries of adaptation to Peru’s diverse ecosystems. Introduced by Spanish colonizers in the 16th century, they likely originated from breeds such as the white Celtiberian and the Castilian of Extremadura—referred to by chroniclers of the time as “Granada,” “Murcia,” and “Málaga” types [1]. Although PCGs are not officially recognized as a formal breed in Peru, they represent a cohesive genetic resource that has diversified in response to the country’s contrasting ecological zones, ranging from arid coastal valleys to high Andean terrains and tropical dry forests.

The regional environments of Peru have shaped goat populations through both natural and human selection. For instance, goats in the Ancash region are raised under extensive grazing systems on hills and mountains, often alongside sheep and cattle. Two systems predominate: small-scale family herds (30 goats on average) for household milk use, and larger herds (150 goats on average) for commercial dairy production [2]. In the Ica region, a desert area with less than 600 mm of annual rainfall, Creole goat farming is common in the Andean steppe zone [3]. These goats are primarily raised for cheese production within extensive management systems [4]. In the Lima region, goat farming occurs in coastal valley and hill ecosystems, using pasture and crop residues [5,6], mainly in small family systems focused on milk and cheese for local markets, with some adoption of management practices [7]. Goat production in the Tumbes and Piura regions occurs within the dry tropical forest ecosystem, which is marked by strong seasonality, high temperatures, and poor soils [8]. Traditional extensive systems based on communal grazing lands and native vegetation are predominant in Peru [9]. Creole goats in these regions show resilience, longevity, and tolerance to harsh conditions [1,10]. In the Lambayeque region, goats are mainly concentrated in the Olmos district (89.4%) near the Piura border and face similar challenges to those in Tumbes and Piura [11]. Climatic variability—particularly irregular rainfall and high temperatures—limits forage availability, affecting goat nutrition and productivity [12].

Despite their importance, information about the genetic diversity, structure, and inbreeding of PCGs is scarce and is essential for either conservation or sustainable use. Application of genomic tools enables detailed analyses of population structure, inbreeding, effective population size, and runs of homozygosity (ROH) [13,14,15]. In this study, we performed the first comprehensive genomic analysis of six PCG populations across Peru (Ancash, Ica, Lambayeque, Lima, Piura, and Tumbes) using high-density SNP data. We assessed the population structure and identified ROH patterns and islands to reveal genomic regions that are potentially under selection.

## 2. Materials and Methods

### 2.1. Ethics Statement

The sample collection was performed in accordance with the Peruvian National Law No. 30407: “Animal Protection and Welfare”.

### 2.2. Animals and Genotyping Data

**Figure 1:**
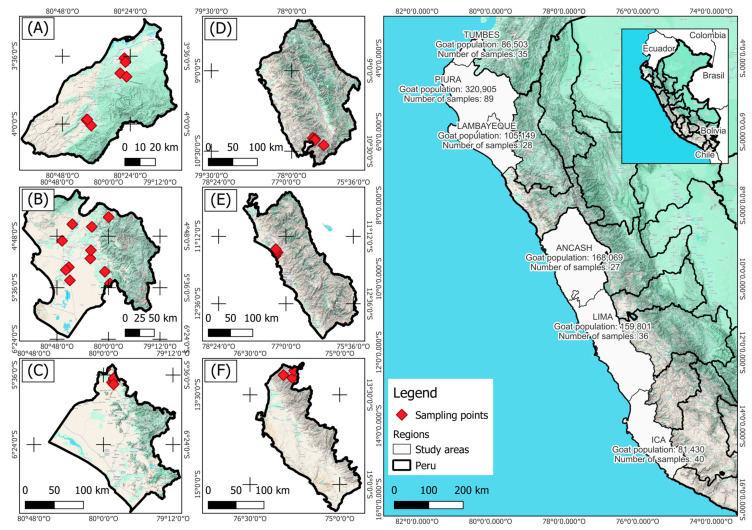
Geographic localization of sampled goat populations across six regions in Peru. Regions: (**A**) Tumbes, (**B**) Piura, (**C**) Lambayeque, (**D**) Ancash, (**E**) Lima, (**F**) Ica. Population estimates are based on data from the most recent Peruvian agricultural census [17].

Ear tissue samples were collected from 106 PCGs across three geographic regions in Peru: Ancash, Ica, and Lima. To enhance geographic representation, genotypes from a publicly available dataset [16] were also included. This dataset comprised samples from the northern regions of Peru: Lambayeque, Tumbes, and Piura. For the present study, both datasets were merged and used in all subsequent analyses (Figure 1).

Only adult goats were sampled, and a maximum of five goats per herd were randomly selected to reduce the likelihood of sampling closely related animals. Herd owners provided information on animal relatedness and origin. Genotyping was performed using the Illumina GGP Goat 70K SNP chip at Neogen Geneseek (Lincoln, NE, USA). This platform includes 67,088 SNP markers distributed across 29 autosomes and two sex chromosomes.

### 2.3. Quality Control and Data Filtering

Quality control was performed using PLINK v1.90 software [18,19], with criteria applied separately for each analysis. For principal component (PCA) and fixation index (FST) analyses, SNPs with a call rate per individual and per marker below 0.95, non-autosomal location, or undefined genomic position were excluded. A minor allele frequency (MAF) lower than 0.05 and a Hardy–Weinberg equilibrium (HWE) *p*-value less than 10^−6^ were applied as a filter criterion to retain markers that are segregating in the population and remove possible sample errors, respectively. Additionally, linkage disequilibrium (LD) pruning was conducted using the PLINK parameter, indep 50 5 2, resulting in 27,788 SNPs retained for these analyses. Quality control was performed considering all populations together and SNPs in common.

For inbreeding coefficient estimation and effective population size (Ne) analyses, SNPs were filtered based on call rates below 0.95 (per individual and per marker), an MAF lower than 0.05, HWE, and non-autosomal location, or undefined genomic position. No filters for LD pruning were applied to avoid bias in homozygosity and Ne estimates. Similarly, for LD decay and the consistency of gametic phase analyses, no LD pruning filtering was applied, as these analyses rely on the natural LD patterns across the genome.

For ROH analyses, SNPs with a call rate below 0.95, non-autosomal location, or undefined genomic position were removed. No filters were applied for MAF, HWE, or LD pruning to preserve the integrity of extended homozygosity segments. The same dataset was used for ROH island detection.

After applying quality control procedures tailored to each analysis, the final dataset for most analyses (excluding ROH) included 255 individuals and 48,932 SNPs. ROH-based analyses employed 49,913 filtered SNPs in the dataset. Overall, the Peruvian regions represented in this study were Ancash (*n* = 27), Ica (*n* = 40), Lambayeque (*n* = 28), Lima (*n* = 36), Piura (*n* = 89), and Tumbes (*n* = 35).

### 2.4. Population Structure and Runs of Homozygosity

To investigate the population structure for all PCGs, PCA and FST analyses were carried out. The PCA was performed using PLINK v1.90 software [18,19], which computes eigenvectors and eigenvalues to capture and summarize the genetic variation present within the dataset. This approach enables the identification of patterns of population stratification and clustering based on genetic similarities. Additionally, the genetic differentiation between each pair of populations was evaluated using FST estimation, also assessed using PLINK v1.90 software [18,19].

ROHs were identified for each individual using PLINK v1.90 software [18,19], which employs a sliding window approach to scan each individual’s genotype across marker positions and detect homozygous segments [20]. To define an ROH, specific criteria were applied: (1) a minimum ROH length of 1 Mb; (2) the inclusion of at least 37 consecutive SNPs in an ROH, calculated based on the equation proposed by Lencz et al. [21], where α (the false positive rate for ROHs) was set to 0.05 in this study; (3) a maximum gap of 0.5 Mb between consecutive SNPs; (4) a minimum density of one SNP per 100 kb; (5) a sliding window of 37 SNPs, advancing one SNP at a time across the genome; (6) tolerance for a maximum of one missing genotype and one heterozygous genotype within an ROH to avoid the underestimation of longer ROHs; and (7) a window threshold set to 0.05. The identified ROHs were categorized into five length classes for further analysis: <2 Mb, 2–4 Mb, 4–8 Mb, 8–16 Mb, and >16 Mb.

To identify the genomic regions that were most commonly associated with ROHs, the percentage of occurrences of SNPs in ROHs was calculated by counting the number of times an SNP was detected in those ROHs across individuals. Genomic regions where ROHs were detected in at least 50% plus one of the individuals were identified as ROH islands and subsequently utilized for functional analyses.

Additionally, genomic regions identified as ROH islands were used for functional analysis. Putative candidate genes within identified regions and their adjacent 3 Mb upstream and downstream regions were identified. Gene annotation was performed using the biomaRt package v2.50.3 [22] in R v4.1.1 software [23], based on the *Capra hircus* reference genome assembly (GoatARS1) available in the Ensembl Genome Browser (https://www.ensembl.org, accessed on 4 December 2024).

### 2.5. Population Metrics

Inbreeding coefficients, linkage disequilibrium, effective population size, and the consistency of the gametic phase were conducted to explore the population metrics of the PCGs.

Five different genomic inbreeding measurements were calculated for all goat populations:

(1) The first method ($F_{HOM1}$) relied on the comparison between the observed and expected proportions of homozygous genotypes where the expected value reflects the proportion under Hardy–Weinberg equilibrium, and the observed value is derived from the sample [24], calculated as follows [18,19]:

(1) $$F_{HOM1}=\frac{H_{exp}-H_{obs}}{H_{exp}}$$

where $H_{exp}$ is the expected value for homozygous genotypes, and $H_{obs}$ is the observed value for the homozygous genotypes.

(2) The second method ($F_{GRM}$) evaluated the genotype additive variance by considering the deviation of the observed allele copies from the expected value, adjusted by the allele frequency in the population [25]:

(2) $$F_{GRM}=\frac{{\left[x_{i}-{2p}_{i}\right]}^{2}}{{2p}_{i}\left(1-p_{i}\right)}-1$$

where $x_{i}$ is the number of reference allele copies on the ith SNP, and $p_{i}$ is the reference allele frequency in the population.

(3) The third method ($F_{HOM2}$), similar to the first, also focused on homozygous genotypes but used a different approach that incorporated the relationship between genotype frequencies and allele frequencies.

(3) $$F_{HOM2}=1-\frac{x_{i}\left(2-x_{i}\right)}{{2p}_{i}\left(1-p_{i}\right)}$$

where all parameters were previously defined.

(4) A fourth method ($F_{UNI}$) was applied to assess the correlation between uniting gametes, providing an alternative perspective on inbreeding estimation, using the following model [26]:

(4) $$F_{UNI}=\frac{\left[x_{i}^{2}-{(1+2p}_{i})x_{i}+2p_{i}^{2}\right]}{{2p}_{i}\left(1-p_{i}\right)}-1$$

(5) The fifth method ($F_{ROH}$) calculated the proportion of the genome covered by runs of homozygosity by dividing the total length of ROHs by the length of the autosomal genome [27]:

(5) $$F_{ROH}=\frac{\sum_{i=i}^{n}f\left({ROH}_{i}\right)}{\sum_{j=1}^{A}h(j)}$$

where $f\left({ROH}_{i}\right)$ is the ROH length of individual *i*, *n* is the total number of homozygous genomic regions of each individual, $h\left(j\right)$ is the length of chromosome *j*, and A is the number of autosomal chromosomes (*A* = 29). Also, for each ROH category (<2 Mb, 2–4 Mb, 4–8 Mb, 4–16 Mb, >16 Mb), inbreeding was estimated as the proportion of the total ROH length relative to the total autosomal genome covered by SNP markers in goats.

All the inbreeding coefficients were computed using PLINK v1.9 software [18,19]. To assess correlations between the inbreeding estimates, the cor function available in R v4.1.1 software [23] was used.

LD was calculated using PLINK v1.9 software [18,19]. To assess the decline of LD with increasing marker distance, a binning strategy was applied, calculating the mean LD for distance intervals ranging from 10 to 100 kb in 10 kb increments. Beyond 100 kb, the mean LD was estimated in 100 kb intervals up to a maximum distance of 1000 kb (1 Mb). For this preliminary analysis, each bin included in the study was required to contain a minimum of 50 pairwise markers to ensure a reliable estimation of the binned LD average.

The estimation of *Ne* was conducted using genomic data and analyzed through the relationship between LD variance and *Ne*, applying the following formula [28]:

(6) $${Ne}_{\left(t\right)}=\frac{1}{\left(4f(c_{t})\right)}\left(\frac{1}{Ε\left[r^{2\vee c_{t}}\right]}-\alpha \right)$$

where *Ne* represents the effective population size in generation *t*, $c_{t}$ denotes the recombination rate corresponding to the physical distance between markers, α is the probability of mutation occurrence, and $r^{2}$ is the measure of LD.

The consistency of the gametic phase was assessed by calculating the square root of the LD values and applying the sign derived from the disequilibrium (D) metric, following the same approach used for LD estimation. The D values were obtained using the –dprime-signed option in PLINK v1.90 software [18,19]. Subsequently, the consistency of the gametic phase between each pair of PCG groups was estimated as the Pearson correlation coefficient (*r*) of the signed-squared-root values. The decay of gametic phase consistency over distance was evaluated using the same bins described previously for LD.

## 3. Results

### 3.1. Population Structure and Runs of Homozygosity

**Figure 2:**
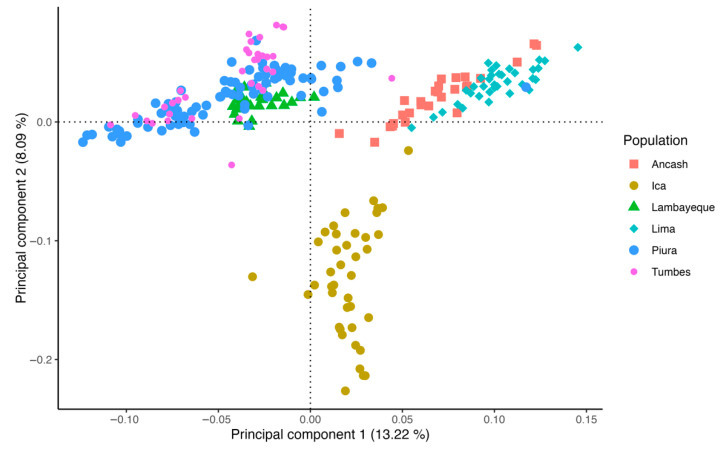
Principal component analysis of Peruvian Creole goat groups.

The PCA of Peruvian Creole goat populations from different regions revealed distinct genetic clusters, suggesting population differentiation. The first principal component accounted for 13.22% of the genetic variance, while the second principal component explained 8.09% (Figure 2). The Lambayeque, Piura, and Tumbes clusters (located in northern Peru) showed moderate overlap, suggesting genetic similarity or shared ancestry among them. The Ica cluster exhibited clear separation from the other groups along the second principal component. Notably, Lima and Ancash clusters were closely positioned along the first principal component, implying possible historical gene flow or genetic relatedness. The spatial distribution of individuals within the PCA plot highlights a moderate genetic structure across the studied populations, likely reflecting the influence of breeding practices, environmental adaptation, and geographical isolation. Additionally, pairwise FST among the PCG populations showed low values ranging from 0.01 to 0.03, indicating low genetic differentiation (Supplementary Table S1).

**Figure 3:**
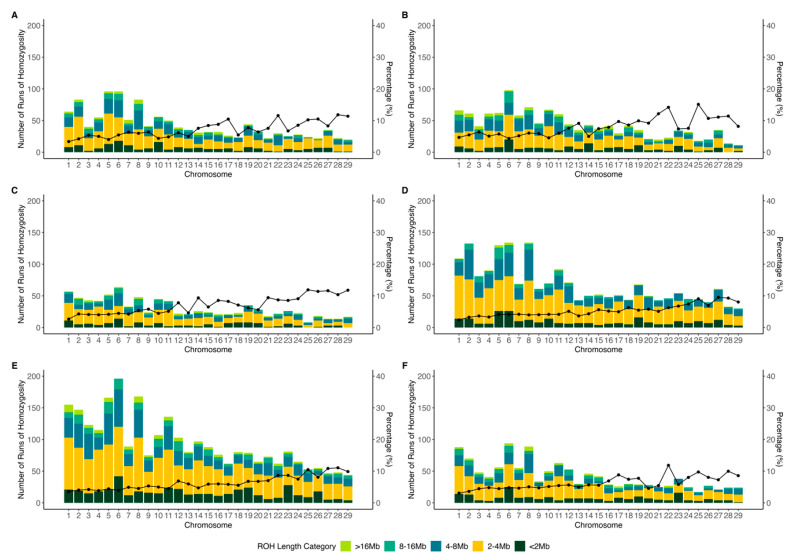
Distribution of runs of homozygosity (ROHs) across chromosomes in Peruvian Creole goats for studied groups: (**A**) Ancash; (**B**) Ica; (**C**) Lambayeque; (**D**) Lima; (**E**) Piura; (**F**) Tumbes.

Figure 3 and Supplementary Table S3 present the distribution of ROH across chromosomes in PCGs. A total of 1238, 1226, 878, 1977, 2740, and 1229 ROH counts were found for the Ancash, Ica, Lambayeque, Lima, Piura, and Tumbes populations, respectively. Shorter ROH segments (<2 Mb, 2–4 Mb, and 4–8 Mb) were the most abundant across all populations, accounting for average proportions of 15.49%, 47.52%, and 24.07%, respectively. In contrast, longer ROH segments (8–16 Mb and >16 Mb) were considerably less frequent, representing only 8.66% and 4.25% on average.

The ROH distribution across chromosomes revealed that chromosome 6 consistently exhibits higher ROH counts in all populations. In the Piura population, for instance, this chromosome harbored 201 ROH segments, accounting for 7.34% of the total ROHs identified. Similar trends were observed in the other populations, where chromosome 6 accounted for 7.75% (Ancash), 7.99% (Ica), 7.29% (Lambayeque), 6.78% (Lima), and 7.65% (Tumbes) of their respective total ROH counts.

The analysis of ROH percentage coverage revealed distinct patterns across chromosomes and populations. Chromosomes 22, 25, and 28 exhibited the highest ROH percentage of coverage across all populations, with values of 10.21%, 11.08%, and 10.66%, respectively. The Ancash, Ica, and Lambayeque populations showed the highest overall ROH coverage across chromosomes, averaging 7.17%, 7.97%, and 7.09%, respectively. Conversely, Lima, Piura, and Tumbes showed lower ROH percentage coverage across several chromosomes with average values of 5.39%, 6.38%, and 6.29%. Piura exhibited consistently moderate to high ROH coverage through all chromosomes. Furthermore, chromosomes 1 through 18 displayed the lowest ROH percentages, particularly in Lambayeque, Lima, and Tumbes. The variability in ROH patterns among populations suggested differences in demographic history, favoring different selective pressures on these populations.

**Figure 4:**
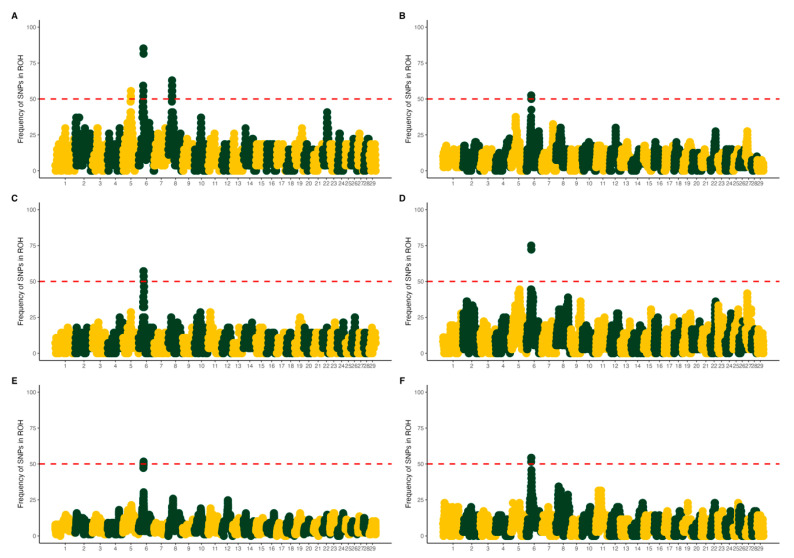
Manhattan plots of ROH islands for studied groups: (**A**) Ancash; (**B**) Ica; (**C**) Lambayeque; (**D**) Lima; (**E**) Piura; (**F**) Tumbes. Each data point represents a SNP, plotted according to its genomic position (*x*-axis: chromosomes 1–29) and its frequency of occurrence within ROH segments (*y*-axis). SNPs are colored yellow for odd-numbered chromosomes and green for even-numbered chromosomes. The red dashed horizontal line indicates the frequency threshold used to define SNPs as part of ROH islands.

Figure 4 shows six Manhattan plots (A–F) depicting the frequency of SNPs within ROHs across chromosomes. Notably, chromosome 6 exhibited peaks in SNP density for all PCG populations, suggesting that the genomic region was under selection pressure in all populations. Table 1 shows the details of runs of homozygosity hotspots detected in chromosome 6.

### 3.2. Population Metrics

The averages of inbreeding coefficients estimated for each population and method are shown in Table 2. Ica displayed the highest average inbreeding coefficient based on the genomic relationship matrix (FGRM = 0.07), whereas Ancash consistently exhibited the highest overall inbreeding levels across multiple metrics (FHOM1 = 0.06, FHOM2 = 0.07, FUNI = 0.06). In contrast, Lambayeque and Piura showed comparatively lower values (0.04) for the same indices. All populations presented low FROH values, suggesting a limited presence of long runs of homozygosity under the thresholds applied.

**Figure 5:**
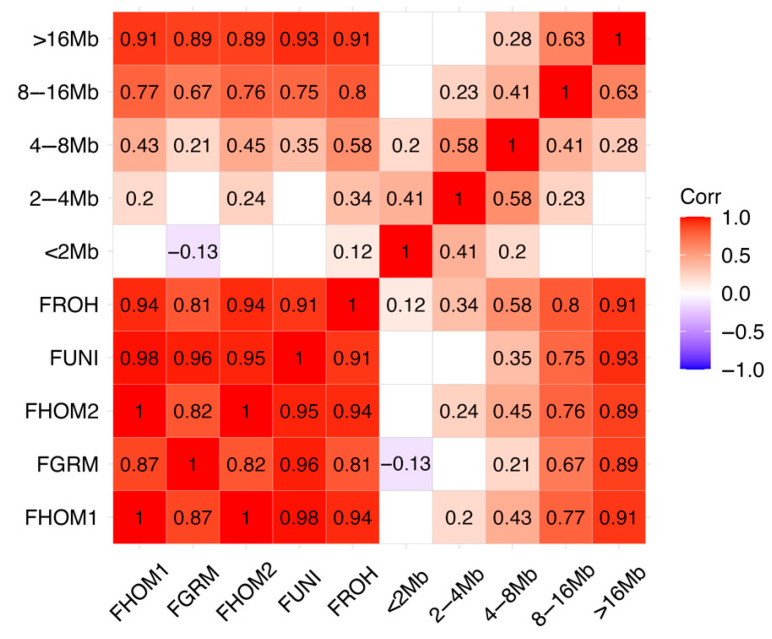
Significant correlations (*p* < 0.05) among inbreeding estimation methods.

Notably, strong positive correlations are observed among most inbreeding estimation methods (Figure 5). The correlations between methods ranged from a minimum of 0.81 (between FROH and FGRM) to a maximum of 1 (between FHOM1 and FHOM2), indicating a high level of agreement among these approaches.

Larger ROH segments, particularly those greater than 16 Mb, exhibited higher correlation (*p* < 0.05) with inbreeding estimates, suggesting that longer homozygous segments are more strongly associated with inbreeding levels as shown in the other metrics. In contrast, smaller ROH segments (<2 Mb to 4–8 Mb) exhibit lower correlations.

**Figure 6:**
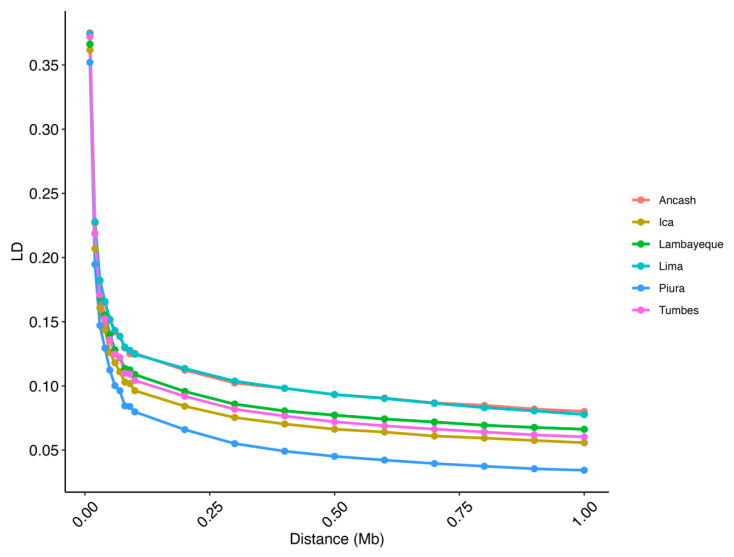
Average linkage disequilibrium (LD) at given distances for the studied goat populations.

At short distances (0.01 Mb), the average LD values were ~0.37 across all populations. As the distance increases, LD values decrease rapidly, with an average of ~0.11 at 0.1 Mb and stabilizing at around 0.08–0.06 beyond 0.25 Mb. Notably, the Piura population shows the lowest LD values across larger distances, reaching 0.03 at 1 Mb. In contrast, a higher LD value at longer distances was observed for the other populations, with values ranging from 0.06 to 0.08 at 1 Mb (Figure 6).

**Figure 7:**
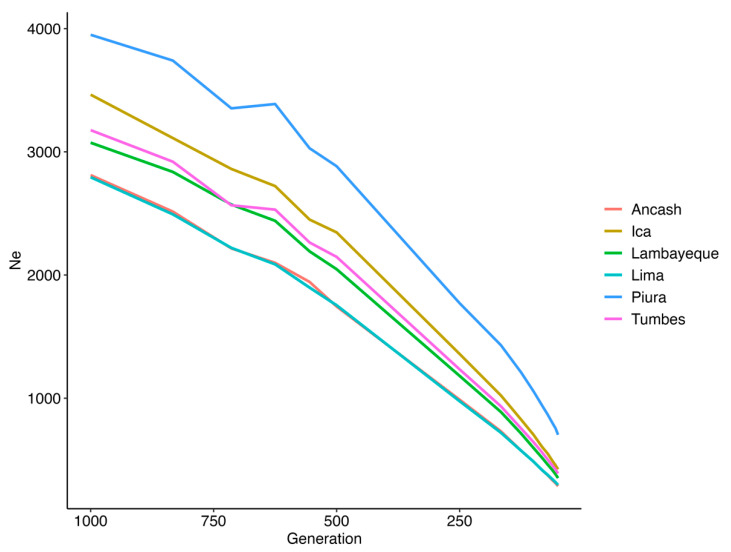
Effective population size (Ne) over the last 1000 generations.

Figure 7 illustrates the average Ne over the past 1000 generations for the different PCG datasets. The results reveal a consistent decline in Ne across all datasets, with varying degrees of reduction. The most recent estimates of Ne, corresponding to generation 50, reveal variation across the six Peruvian Creole goat populations analyzed. The Piura population exhibited the highest Ne, with a value of 703, indicating a comparatively larger and more genetically diverse breeding population in recent generations. This was followed by Ica (Ne = 423) and Tumbes (Ne = 389). In contrast, the populations from Lambayeque, Lima, and Ancash showed relatively smaller effective population sizes, with Ne values of 352, 296, and 286, respectively.

**Figure 8:**
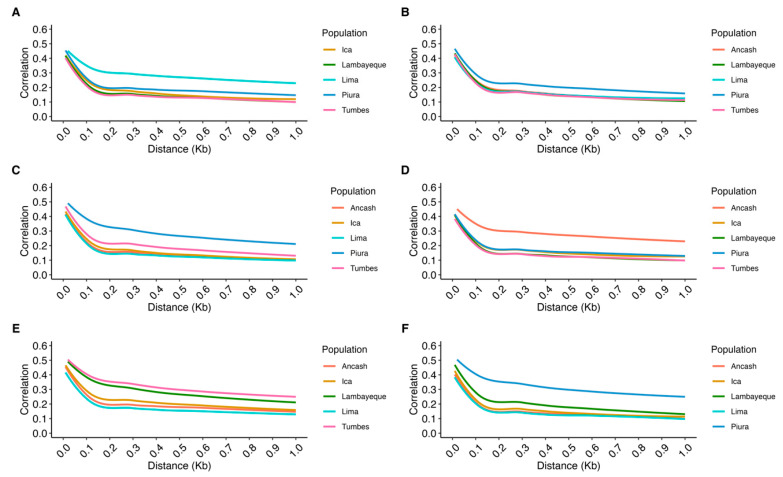
Consistency of the gametic phase in Peruvian Creole goat populations. Each panel (**A**–**F**) shows the decay of correlation in the gametic phase between the focal population and the others: (**A**) Ancash; (**B**) Ica; (**C**) Lambayeque; (**D**) Lima; (**E**) Piura; (**F**) Tumbes.

Figure 8 and Supplementary Table S2 show the consistency of the gametic phase across different genomic distances (in megabases, Mb) for the six PCG populations. The highest gametic phase consistency correlations, assessed at a 10 kb of distance between SNP pairs, were observed between the Piura and Lambayeque populations (r = 0.64), as well as between Piura and Tumbes (r = 0.64). In contrast, the lowest correlations were detected at a 1 Mb of distance between SNPs, particularly between Lima and Lambayeque (r = 0.10) and between Lima and Tumbes (r = 0.10), indicating a lower consistency in the gametic phase.

## 4. Discussion

### 4.1. Population Structure and Runs of Homozygosity

The PCA results reveal a genetic structure among PCG populations, with three distinct clusters indicating genetic differentiation and geographical relationship. The observed genetic similarities among the northern populations (Lambayeque, Piura, and Tumbes) suggest potential historical gene flow or shared ancestry, possibly due to past breeding practices or geographical proximity. In contrast, the clear separation of the southern Ica population highlights significant genetic divergence, which could be attributed to geographical isolation or distinct selection pressures. The proximity of the Ancash and Lima populations in the PCA space suggests a closer genetic relationship, potentially influenced by similar breeding strategies and/or environmental factors. The genetic structure observed reflects the influence of geographic barriers, environmental adaptation, and limited gene flow among regions, particularly for the Ica population which is isolated in an Andean steppe environment (Figure 1). This pattern is consistent with the evolutionary history of domestic goats in Peru, where Spanish colonial introductions diversified through adaptation to local conditions while maintaining overall genetic continuity.

These findings align with previous continental-scale studies that have reported moderate levels of genetic diversity and clear population structuring among Creole goat populations across Latin America [29,30]. Ginja et al. [29] highlighted the moderate genetic differentiation among populations, with regional clustering patterns influenced by geographical proximity and historical breeding practices, whereas Aguirre-Riofrio et al. [30] focused on the “Chusca Lojana,” from Ecuador which exhibited unique genetic characteristics, which underscores the impact of geographical isolation and localized breeding on genetic differentiation.

The low FST values observed in this study (0.01–0.03) require careful interpretation when compared to continental-scale studies. For instance, Ginja et al. [29] reported higher FST values between Creole goat populations from different countries across the Americas using 21 SSR markers. However, several factors explain this apparent discrepancy. First, the geographic scale differs significantly: our study examines populations within a single country (Peru), while Ginja et al. [29] compared populations across different countries and continents. Within-country populations are expected to show lower genetic differentiation due to more recent shared ancestry and greater potential for gene flow. Second, the marker systems differ substantially: our study employed high-density SNP data (70K chip) while Ginja et al. used 21 SSR markers. The different marker densities and types can influence FST estimates. Third, the relatively large standard deviations in our FST estimates limit the strength of our conclusions, suggesting that while differentiation exists, it is moderate rather than pronounced. Despite these low FST values, the clear population structure observed in the PCA indicates meaningful genetic differentiation that has biological and conservation relevance.

Moreover, it is important to clarify that the Peruvian Creole goat should be considered as a single population with regional differentiation rather than six distinct breeds. While these animals are not officially recognized as a formal breed in Peru, they represent a cohesive genetic resource that has diversified into regional ecotypes or variants adapted to different environmental conditions across Peru’s diverse landscapes. The populations studied represent local adaptations of the same foundational genetic stock introduced during Spanish colonization, which have undergone regional differentiation through natural and artificial selection pressures specific to each geographic area. Based on the observed results, it is feasible to conduct genealogical, phenotypic, and productive documentation of the population with the aim of enabling the government to provide racial recognition to this population.

Although some regions such as Ancash and Lambayeque are marginally represented in the dataset (*n* = 27 and *n* = 28, respectively), this imbalance was primarily due to logistical constraints and limited accessibility during the sampling period. Despite these limitations, samples from these regions were included to ensure broader geographic coverage and to capture at least a partial view of their genetic diversity. We acknowledge this limitation and recommend that future studies aim for more balanced sampling to enhance the strength of population-level comparisons.

The analysis of ROH patterns offers valuable insights into the demographic and genetic history of populations [31,32]. ROHs arise when both parents convey identical chromosomal segments inherited from a common ancestor to their descendants, leading to regions of homozygosity [15]. Typically, short ROHs reflect ancient inbreeding, as recombination over successive generations tends to fragment these segments [33]. Conversely, the presence of long ROHs is indicative of recent inbreeding, suggesting a lack of recombination due to recent shared ancestry [14].

The distribution of ROHs across chromosomes in the PCGs reveals population-specific differences in homozygosity patterns, reflecting varying demographic histories and selective pressures. The high frequency of short ROH segments (2–4 Mb), accounting for an average of 47.52% across all populations, suggests a higher degree of ancestral inbreeding [33]. In contrast, longer ROH segments (>16 Mb), which are indicative of more recent inbreeding [34,35], are less frequent across all populations (4.25%). This pattern—short ROHs being more common and long ROHs are rare—supports the idea that most inbreeding events in these populations are historically distant rather than recent.

ROH island analysis was performed by evaluating the frequency of SNPs present within ROHs across individuals in each population. ROH islands were defined as genomic regions where the percentage of individuals carrying a given SNP within an ROH exceeded the threshold of 50% plus one individual. Notably, chromosome 6 harbored ROH islands in all populations, indicating a region of shared homozygosity among the PCG populations. This finding suggests the presence of a conserved functional region or shared selection pressure across populations. Ancash exhibited three ROH islands located on chromosomes 5, 6, and 8, while the overall ROH island landscape across the six PCG populations exhibited partially overlapping patterns of genomic homozygosity. The consistent identification of ROH islands, particularly on chromosome 6, highlights potential regions under selection that could reveal selection signatures in PCG populations.

Within these ROH islands on chromosome 6, several candidate genes were identified that are likely involved in economically and biologically important traits. For instance, G protein-regulated inducer of neurite outgrowth 3 (*GPRIN3*) has been linked to prolificacy and temperament traits in sheep, supporting its role in reproductive and behavioral phenotypes [36,37]. Also located within this region, family with sequence similarity 13 member A (*FAM13A*), a gene that inhibits goat intramuscular adipocyte differentiation via the RIG-I signaling pathway [38], and is associated with somatic cell count in Jersey cattle [39], was also located. Genes from the HERC family of ubiquitin protein ligase family members 3, 5, and 6 (*HERC3*, *HERC5*, and *HERC6*) have been associated with four biological pathways in sheep, including the immune system, ubiquitination and proteasome degradation, Class I MHC mediated antigen processing, and adaptive immune system pathways [40]. These genes have also been reported to interact with prolactin to regulate key milk proteins such as β-casein [41], and have been proposed as novel candidate genes for milk protein content due to their previously reported associations with protein and fat percentages in milk [42]. Furthermore, secreted phosphoprotein 1 (*SPP1*), a gene involved in maternal–fetal angiogenesis, calcium metabolism, and immune function by enhancing T-helper cell responses [43,44], was found within the chromosome 6 shared ROH island. Other key regions include the well-characterized cluster comprising *LAP3*, *MED28*, *FAM184B*, *DCAF16*, *NCAPG*, and *LCORL* associated with carcass weight and growth [45,46,47] and milk production traits in cattle [48,49].

The presence of these genes within ROH islands suggests that these genomic regions may play crucial roles in local adaptation and/or economically important traits. The clustering of ROHs within these regions further supports the hypothesis of selection acting on specific loci. Gene enrichment analysis was performed for the candidate genes located within ROH islands; however, no statistically significant results were obtained. This may be due to the limited number of genes identified or the lack of functional annotations available for these genes, which may constrain the power to detect meaningful enrichment. Further functional validation and association studies are necessary to elucidate the phenotypic impacts of these candidate genes.

### 4.2. Population Metrics

The low average values of FROHs across all populations suggest a limited presence of recent inbreeding events. This aligns with previous findings in indigenous goat populations managed under extensive systems, where mating is often unregulated but populations are large enough to maintain genetic variability [50,51]. In comparison, higher inbreeding values have been reported in commercial or endangered breeds due to population bottlenecks and selective breeding [13,52].

The high correlations among different inbreeding estimation methods suggest that these approaches effectively capture genomic inbreeding patterns in PCG populations. Notably, the strong association between larger ROH segments (>16 Mb) and inbreeding coefficients indicates that recent inbreeding events contribute significantly to the overall inbreeding levels. Conversely, weaker correlations observed with shorter ROH segments (<2 Mb) suggest that these smaller segments may result from historical recombination rather than recent consanguinity. The strong positive correlations observed between the inbreeding metrics, particularly between FHOM1 and FUNI (r = 0.98), suggest that these estimators capture similar aspects of genome-wide homozygosity, as previously reported in studies [53,54].

Analysis of LD in PCG populations revealed patterns that are expected in genetically diverse and historically unmanaged populations [55,56]. LD values were highest at short distances between markers (average ~0.37 at 10 kb) and decreased rapidly with increasing distance, stabilizing between 0.08 and 0.06 at 0.5 Mb. These patterns are consistent with those observed in indigenous goat populations [50,57]. Among the PCG populations, Piura showed the fastest LD decline and the lowest LD values at long distances, reaching 0.03 at 1 Mb. This suggests a higher historical recombination rate and greater diversity, likely influenced by admixture or continuous gene flow from neighboring regions, as supported by the high consistency of the gametic phase with Lambayeque and Tumbes (Supplementary Table S2). In contrast, the Ancash and Lima populations showed comparatively higher LD levels at longer distances (0.06–0.08 at 1 Mb), suggesting possible limited gene flow or structured breeding that preserved larger linkage.

Similar patterns have been reported in studies of criollo cattle in Latin America. For example, Bejarano et al. [58], Caivio-Nasner et al. [59], and Bottani [60] found a moderate to rapid decline in LD in creole cattle breeds from Colombia and Bolivia, reflecting their adaptation to local environments and a minimal influence of commercial breeding. The low LD levels are consistent with less intensively selected populations, such as PCG populations [61]. Furthermore, Monau et al. [55] showed that indigenous African goats also exhibit a rapid decline in LD, similar to that observed in Piura. These results contrast with those observed in intensively farmed commercial breeds, which often exhibit a slower decline in LD due to artificial selection and a smaller effective population size [55,62]. This study presents one of the first analyses of linkage disequilibrium patterns in Creole goats from Latin America, providing valuable information for comparative studies with other Creole breeds.

The historical trajectory of Ne across the six populations of PCGs reveals a consistent and marked decline (Figure 7). According to Frankham et al. [63], an Ne of 50 is insufficient to prevent fitness loss due to inbreeding. To limit this loss to 10% over five generations, an Ne of at least 100 is required. Furthermore, maintaining long-term evolutionary potential demands an Ne of no less than 1000.

Ancash (Ne = 286) and Lima (Ne = 296) exhibit the lowest recent Ne values among the PCG populations. While these values do not currently indicate a risk of genetic erosion, they highlight the importance of establishing monitoring indicators for inbreeding and maintaining genetic diversity. Tumbes (Ne = 389) and Lambayeque (Ne = 352) show moderately higher Ne values, which remain above thresholds typically associated with genetic risk (e.g., Ne < 150), but still merit continued observation. In contrast, Piura (Ne = 703) and Ica (Ne = 423) maintain relatively higher effective population sizes, indicating more stable breeding populations.

Comparisons with other local and regional goat breeds support the relevance of these findings. For instance, Kichamu et al. [64], using pedigree-based estimators, reported much lower Ne values for African indigenous breeds such as Galla (Ne = 5.19) and Small East African goats (Ne = 4.77), reflecting severe genetic bottlenecks in those populations. Similarly, Mandal et al. [65] reported an Ne of 52.65 for the Jamunapari goat in India, though the periodic introduction of unrelated breeding males helped maintain inbreeding levels that were below critical thresholds. In Iranian Markhoz goats, Rashidi et al. [66] estimated Ne values of 84 and 69 using different pedigree-based approaches. In light of these comparisons, the PCG—despite showing declining trends—still maintains relatively moderate Ne levels, particularly in regions such as Piura.

Supplementary Table S2 shows that the highest gametic phase (at 10 kb) was observed among northern populations, particularly Piura–Tumbes (r = 0.63), suggesting that most of their alleles are phased similarly. In contrast, a low consistency of the gametic phase was observed between northern and central populations, such as Tumbes–Lima, with correlations as low as 0.10 at 1 Mb. In practice, this low level of consistency of the gametic phase could indicate that these populations cannot be reliably combined within the same genomic evaluation system, as the phases of their alleles are mostly opposite.

The gametic phase in PCG populations was generally low compared to values reported in specialized goat breeds [67,68,69]. For example, Vermette et al. [67] reported a maximum phase correlation of ~0.50 between Alpine and Saanen goats at short distances. Brito et al. [69] observed a greater gametic phase between Australian and Canadian Boer goats, with values close to 0.70 for short distances (<0.02 Mb), which was expected given their shared breed origin despite geographic separation. A study by Teissier et al. [68] on the Alpine breed found a very high gametic phase between countries (France-Italy: r = 1.0 to <1 kb) that gradually decreased with distance (r = 0.67 at 500–1000 kb), while transcontinental comparisons, such as that of Canada–France, showed a steeper decline (r = 0.11 at 1 Mb).

### 4.3. Conservation and Breeding Implications

Based on the genetic structure and diversity patterns observed in this study, we recommend implementing a regional approach to conservation and breeding programs for Peruvian Creole goats. The PCA results and genetic differentiation patterns suggest that three main conservation units should be established: (1) Northern Region Program (Lambayeque, Piura, and Tumbes): These populations show genetic similarity and share similar environmental conditions in the dry tropical forest ecosystem. A joint conservation program would be most effective, with emphasis on maintaining the high genetic diversity observed in Piura while preventing further genetic erosion in Lambayeque and Tumbes. (2) Central Region Program (Ancash and Lima): These populations cluster together in the PCA and share coastal and highland environments. A coordinated program should focus on maintaining genetic diversity while addressing the relatively lower effective population sizes observed in these regions. (3) Southern Region Program (Ica): This population shows the most genetic distinctiveness and is adapted to the Andean steppe conditions. A specific conservation program is needed to preserve this unique genetic resource while preventing further isolation that could lead to inbreeding depression. However, these regional programs should maintain some level of gene flow between regions to prevent further genetic differentiation while preserving local adaptation. The relatively low FST values suggest that controlled introduction of breeding animals among regions could be beneficial for maintaining overall genetic diversity without compromising local adaptation.

These findings offer valuable opportunities to design genomic-based breeding programs aimed at supporting conservation strategies, strengthening population management, and optimizing the selection of males and females for PCG utilization and promotion in Peru.

## 5. Conclusions

The current study offers the first genome-wide assessment of inbreeding metrics, linkage disequilibrium, effective population size, consistency of the gametic phase, and runs of homozygosity in PCGs from six geographic regions in Peru. The analysis revealed moderate genetic structuring, with northern populations (Tumbes and Piura) exhibiting genetic similarity and Ica showing distinct divergence. Inbreeding levels were generally low, aligning with patterns observed in other indigenous goat populations managed under extensive systems. LD patterns indicated rapid decay, particularly in Piura, suggesting higher historical recombination rates and genetic diversity. Effective population size estimates showed a declining trend across all populations, with Piura maintaining relatively higher Ne values, highlighting the need for conservation efforts to prevent further genetic erosion. ROH analysis identified population-specific differences, with Piura exhibiting the highest number of ROH segments. Notably, ROH islands on chromosome 6 were identified across all populations, encompassing genes associated with reproduction, immunity, and production traits. These findings provide a foundational understanding of the population structure and diversity of PCG populations.

## Figures and Tables

**Table 1:** Details of runs of homozygosity hotspots detected in chromosome 6.

Chr	Start (bp)	End (bp)	Length	Gene	Description
6	36,070,368	36,072,710	2342	*GPRIN3*	GPRIN family member 3
6	36,205,904	36,207,478	1574	*TIGD2*	Tigger transposable element derived 2
6	36,424,597	36,551,359	126,762	*FAM13A*	Family with sequence similarity 13 member A
6	36,572,520	36,707,017	134,497	*HERC3*	HECT and RLD domain containing E3 ubiquitin protein ligase 3
6	36,593,698	36,594,279	581	*NAP1L5*	Nucleosome assembly protein 1 like 5
6	36,759,209	36,761,094	1885	*PYURF*	PIGY upstream open reading frame
6	36,765,648	36,813,335	47,687	*HERC5*	HECT and RLD domain containing E3 ubiquitin protein ligase 5
6	36,819,857	36,872,364	52,507	*HERC6*	HECT and RLD domain containing E3 ubiquitin protein ligase family member 6
6	36,967,148	36,981,000	13,852	*PPM1K*	Protein phosphatase, Mg2+/Mn2+ dependent 1K
6	36,987,612	37,118,958	131,346	*ABCG2*	ATP binding cassette subfamily G member 2 (JR blood group)
6	37,126,098	37,193,068	66,970	*PKD2*	Polycystin 2, transient receptor potential cation channel
6	37,213,669	37,221,650	7981	*SPP1*	Secreted phosphoprotein 1
6	37,364,698	37,368,465	3767	*MEPE*	Matrix extracellular phosphoglycoprotein
6	37,397,003	37,414,860	17,857	*IBSP*	Integrin binding sialoprotein
6	37,669,173	37,694,927	25,754	*LAP3*	Leucine aminopeptidase 3
6	37,696,130	37,702,512	6382	*MED28*	Mediator complex subunit 28
6	37,708,219	37,826,187	117,968	*FAM184B*	Family with sequence similarity 184 member B
6	37,846,165	37,848,349	2184	*DCAF16*	DDB1 and CUL4 associated factor 16
6	37,858,170	37,903,004	44,834	*NCAPG*	Non-SMC condensin I complex subunit G
6	37,905,295	38,068,616	163,321	*LCORL*	Ligand dependent nuclear receptor corepressor like

**Table 2:** Average inbreeding coefficient for each population and inbreeding metric ^1^.

Inbreeding Metric	Ancash	Ica	Lambayeque	Lima	Piura	Tumbes
FHOM1	0.06	0.05	0.04	0.05	0.04	0.05
FGRM	0.05	0.07	0.04	0.03	0.04	0.04
FHOM2	0.07	0.04	0.04	0.05	0.04	0.05
FUNI	0.06	0.06	0.04	0.04	0.04	0.05
FROH	1.04× 10^−4^	7.60 × 10^−5^	6.66 × 10^−5^	9.35 × 10^−5^	6.13 × 10^−5^	7.14 × 10^−5^
<2 Mb	4.80 × 10^−6^	3.09 × 10^−6^	3.35 × 10^−6^	5.35 × 10^−6^	3.63 × 10^−6^	4.24 × 10^−6^
2–4 Mb	2.41 × 10^−5^	1.49 × 10^−5^	1.68 × 10^−5^	3.26 × 10^−5^	1.64 × 10^−5^	1.96 × 10^−5^
4–8 Mb	2.58 × 10^−5^	1.71 × 10^−5^	1.57 × 10^−5^	3.20 × 10^−5^	1.61 × 10^−5^	1.66 × 10^−5^
8–16 Mb	1.86 × 10^−5^	1.50 × 10^−5^	1.47 × 10^−5^	1.60 × 10^−5^	1.08 × 10^−5^	1.40 × 10^−5^
>16 Mb	3.09 × 10^−5^	2.59 × 10^−5^	1.60 × 10^−5^	7.54 × 10^−6^	1.44 × 10^−5^	1.70 × 10^−5^

^1^ FROH and ROH length category values are shown in scientific notation.

## Data Availability

The data that support the findings of this study are openly available at Zenodo under record number 16750183 at https://zenodo.org (accessed on 27 June 2025).

## Supplementary Materials

The following supporting information can be downloaded at https://www.mdpi.com/article/10.3390/ani15172577/s1: Table S1: Mean and Standard deviation of FST and FST selected for comparisons between six Peruvian Creole goat populations; Table S2: Consistency of the gametic phase based on Pearson correlation across Peruvian Creole goats; Table S3: Counts of ROH length across goat groups.

## Abbreviations

The following abbreviations are used in this manuscript:

PCG	Peruvian Creole goats
SNP	Single nucleotide polymorphism
ROH	Runs of homozygosity
PCA	Principal component analysis
FST	Fixation index
MAF	Minor allele frequency
HWE	Hardy–Weinberg equilibrium
LD	Linkage disequilibrium
Ne	Effective population size
GPRIN3	GPRIN family member 3
TIGD2	Tigger transposable element derived 2
FAM13A	Family with sequence similarity 13 member A
HERC3	HECT and RLD domain containing E3 ubiquitin protein ligase 3
NAP1L5	Nucleosome assembly protein 1 like 5
PYURF	PIGY upstream open reading frame
HERC5	HECT and RLD domain containing E3 ubiquitin protein ligase 5
HERC6	HECT and RLD domain containing E3 ubiquitin protein ligase family member 6
PPM1K	Protein phosphatase, Mg2+/Mn2+ dependent 1K
ABCG2	ATP binding cassette subfamily G member 2 (JR blood group)
PKD2	Polycystin 2, transient receptor potential cation channel
SPP1	Secreted phosphoprotein 1
MEPE	Matrix extracellular phosphoglycoprotein
IBSP	Integrin binding sialoprotein
LAP3	Leucine aminopeptidase 3
MED28	Mediator complex subunit 28
FAM184B	Family with sequence similarity 184 member B
DCAF16	DDB1 and CUL4 associated factor 16
NCAPG	Non-SMC condensin I complex subunit G
LCORL	Ligand dependent nuclear receptor corepressor like
