# Supplementary material for: Genomic Characterization of Peruvian Creole Goats: Insights into Population Structure and Runs of Homozygosity
Source: Animals (Basel). 2025 Sep 2;15(17):2577. doi: 10.3390/ani15172577 (PMC12427317; doi:10.3390/ani15172577)
Supplement: Supplementary file 1 [file animals-15-02577-s001.zip › animals-3755402-supplementary.pdf]

animals

MDPI

Supplementary Table S1. Mean and Standard deviation of FST and FST selected[1] for comparisons between six Peruvian Creole goat's populations.

	FST		FST selected[1]		
**Population comparisons**	**Mean**	**SD**	**N**	**Mean**	**SD**
Ancash vs Ica	0.02	0.04	375	0.22	0.05
Ancash vs Lambayeque	0.02	0.05	479	0.22	0.06
Ancash vs Lima	0.01	0.04	143	0.21	0.04
Ancash vs Piura	0.02	0.04	192	0.22	0.06
Ancash vs Tumbes	0.02	0.05	619	0.22	0.06
Ica vs Lambayeque	0.02	0.05	476	0.22	0.05
Ica vs Lima	0.02	0.05	525	0.22	0.05
Ica vs Piura	0.02	0.04	190	0.22	0.06
Ica vs Tumbes	0.02	0.05	466	0.22	0.05
Lambayeque vs Lima	0.03	0.05	763	0.23	0.06
Lambayeque vs Piura	0.01	0.03	33	0.20	0.03
Lambayeque vs Tumbes	0.01	0.04	242	0.21	0.04
Lima vs Piura	0.02	0.04	339	0.21	0.05
Lima vs Tumbes	0.03	0.06	929	0.23	0.05
Piura vs Tumbes	0.01	0.02	25	0.20	0.02

[1] FST selected: FST values calculated from loci identified as highly differentiated. These are SNPs whose FST values fall outside the range defined by the population-wide mean FST ± 3.5 times the standard deviation ($FST_{Mean} \pm 3.5 * FST_{SD}$). This threshold captures both unusually high and unusually low FST values, which may indicate loci under selection

Supplementary Table S2. Consistency of the gametic phase based on Pearson correlation across Peruvian Creole goat.

Population	Group	Mean	Min	Max	SD
Ancash	Ica	0.24	0.12	0.53	0.11
	Lambayeque	0.22	0.10	0.52	0.11
	Lima	0.34	0.23	0.61	0.10
	Piura	0.27	0.15	0.56	0.11
	Tumbes	0.21	0.10	0.50	0.11
Ica	Lambayeque	0.24	0.11	0.54	0.12
	Lima	0.23	0.13	0.51	0.10
	Piura	0.29	0.16	0.56	0.11
	Tumbes	0.23	0.11	0.55	0.11
Lima	Lambayeque	0.22	0.10	0.51	0.11
	Piura	0.24	0.13	0.52	0.10
	Tumbes	0.21	0.10	0.49	0.10
Lambayeque	Piura	0.36	0.21	0.61	0.11
	Tumbes	0.27	0.13	0.58	0.12
Piura	Tumbes	0.38	0.25	0.63	0.11

Supplementary Table S3. Counts of ROH length across goat groups.

ROH length	Ancash	Ica	Lambayeque	Lima	Piura	Tumbes	ROH percentage
<2Mb	184	178	132	272	462	211	15.49%
2-4Mb	562	518	416	1024	1299	595	47.52%
4-8Mb	314	309	195	522	639	257	24.07%
8-16Mb	114	134	94	132	218	112	8.66%
>16Mb	64	87	41	27	122	54	4.25%
Total	1238	1226	878	1977	2740	1229	100%
